# A Magnetic Bead-Integrated Chip for the Large Scale Manufacture of Normalized esiRNAs

**DOI:** 10.1371/journal.pone.0039419

**Published:** 2012-06-27

**Authors:** Zhao Wang, Huang Huang, Hanshuo Zhang, Changhong Sun, Yang Hao, Junyu Yang, Yu Fan, Jianzhong Jeff Xi

**Affiliations:** 1 Department of Biomedical Engineering, College of Engineering, Peking University, Beijing, China; 2 State Key Laboratory of Transducer Technology, Chinese Academy of Sciences, Shanghai, China; University of Kansas Medical Center, United States of America

## Abstract

The chemically-synthesized siRNA duplex has become a powerful and widely used tool for RNAi loss-of-function studies, but suffers from a high off-target effect problem. Recently, endoribonulease-prepared siRNA (esiRNA) has been shown to be an attractive alternative due to its lower off-target effect and cost effectiveness. However, the current manufacturing method for esiRNA is complicated, mainly in regards to purification and normalization on a large-scale level. In this study, we present a magnetic bead-integrated chip that can immobilize amplification or transcription products on beads and accomplish transcription, digestion, normalization and purification in a robust and convenient manner. This chip is equipped to manufacture ready-to-use esiRNAs on a large-scale level. Silencing specificity and efficiency of these esiRNAs were validated at the transcriptional, translational and functional levels. Manufacture of several normalized esiRNAs in a single well, including those silencing PARP1 and BRCA1, was successfully achieved, and the esiRNAs were subsequently utilized to effectively investigate their synergistic effect on cell viability. A small esiRNA library targeting 68 tyrosine kinase genes was constructed for a loss-of-function study, and four genes were identified in regulating the migration capability of Hela cells. We believe that this approach provides a more robust and cost-effective choice for manufacturing esiRNAs than current approaches, and therefore these heterogeneous RNA strands may have utility in most intensive and extensive applications.

## Introduction

RNA interference (RNAi) is an intrinsic cellular mechanism for mediating gene expression, and has been established as a commonly used tool for gene down-regulation and loss-of-function studies in the life sciences [1]. The siRNA duplex and shRNA are generally chemically synthesized and used for experimental analysis in mammalian cells [2]. Endoribonuclease-prepared siRNA (esiRNA), which is generated by bacterial RNase III digestion of a long dsRNA, is a mixture of siRNA-like molecules of heterogeneous strands that all target the same transcript; these esiRNA thus can produce the same silencing efficiency with a 12-fold lower off-target effect compared to chemically synthesized siRNA [3]–[6]. Accordingly, esiRNA has recently attracted the attention of researchers due to both its reduced off-target effect and cost-effectiveness.

For example, Kittler *et al.* identified over 1,000 new genes required for cell division based on results from their high-throughput cell viability screening [5]. Collinet *et al.* systematically investigated and identified several novel components in the transferrin (TF) and epidermal growth factor (EGF)-related endocytic trafficking [7]. In addition, Fazzio *et al.* demonstrated that esiRNAs are highly effective in RNAi-mediated gene silencing in embryonic stem cell (ESCs), while Tan et al. showed that esiRNAs were able to inhibit HBV replication more efficiently than synthesized siRNAs and tolerated limited target sequence variations without losing inhibitory capacity [8], [9].

The production of esiRNA, however, is a complex process involving multiple steps, including product purification, quantification and normalization. As RNase III enzymes do not completely digest long dsRNAs, the purification step is essential. In addition, normalization of esiRNA prior to transfection is a critical but tedious task. We previously demonstrated the manufacture of hundreds of esiRNAs using a polymer microbead-integrated chip [10]. Here, we report further simplification of the manufacturing process and describe a magnetic bead-integrated chip that is capable of manufacturing hundreds of ready-to-use esiRNAs simultaneously. Furthermore, we present two functional assays using the esiRNAs, which demonstrates their successful production and application in cell function analysis, and illustrates the robustness of this chip.

## Results and Discussion

**Figure 1:**
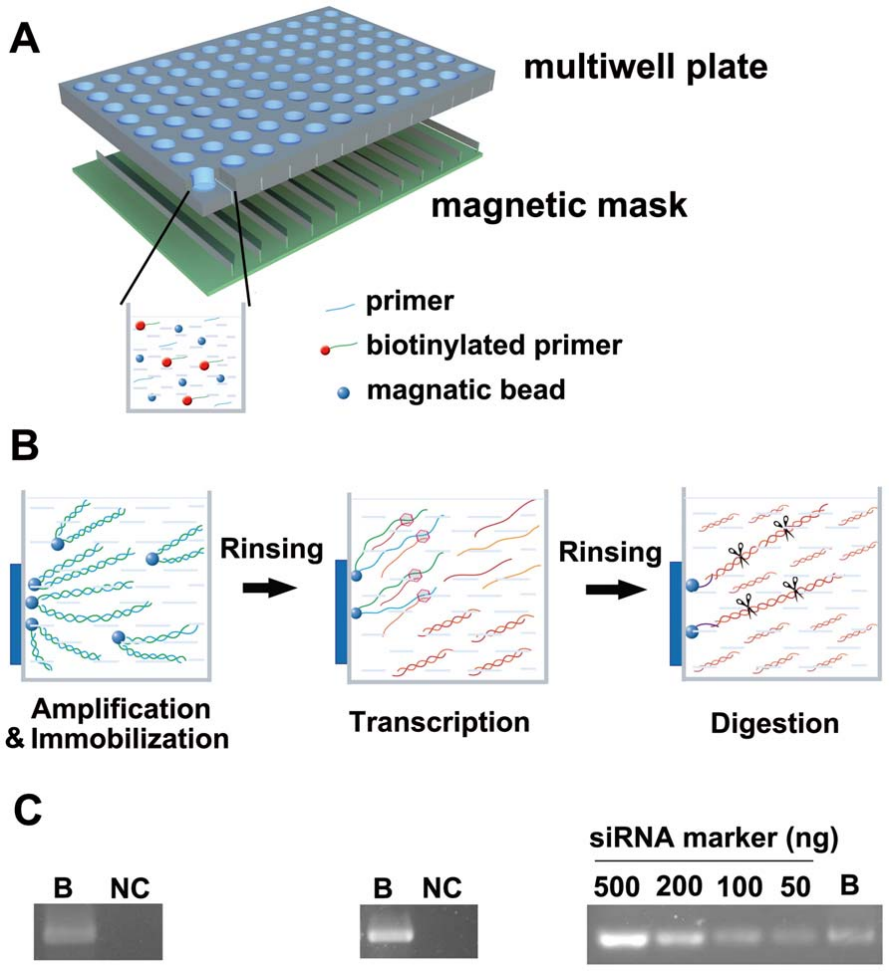
Manufacture of esiRNA by means of a magnetic bead-integrated chip. **A.** The schematic diagram shows the chip composed of a microwell array and magnetic beads coated with streptavidin. **B.** Large-scale manufacture of esiRNAs can be divided into three steps: target amplification and immobilization, transcription, and enzymatic digestion. **C.** Confirmation of PCR reactions using biotinylated or non-biotinylated primers. Amplification products (left), transcription products (middle), or esiRNA products (right) are detected only when biotinylated DNA primers are used. B: biotinylated primers.

The chip contained two components: a microwell array and magnetic microbeads coated with streptavidin (Figure 1A). The utilization of this chip allows the process of large-scale production of esiRNA to be simplified into three main steps: target amplification and immobilization, transcription, and enzymatic digestion (Figure 1B).

**Figure 2:**
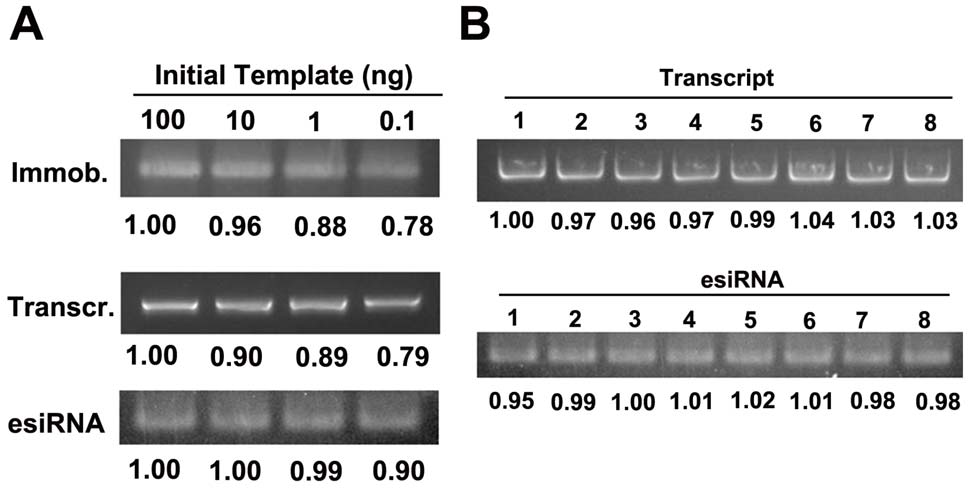
The normalization of esiRNA products. **A.** A range of initial amounts of DNA template were assayed (100, 10, 1, and 0.1 ng, each in 5 µL in volume). After the immobilization and transcription steps, the variation of transcription products was within 20%. The final esiRNA products had a variation of less than 10% when the initial amount of DNA templates was in the range of 0.1–100 ng. **B**. Eight esiRNA products were manufactured in parallel. The standard deviation among these eight products was approximately 3%.

First, a group of linearized DNA fragments, which could either be cloned cDNA fragments or chip-based, chemically-synthesized DNA oligomers, were dispensed into a microwell chip as the PCR template. One of the two PCR primers was biotinylated to facilitate the immobilization of the amplification products on beads after PCR and the subsequent easy separation from the supernatant containing dNTPs, DNA polymerase and buffer solution. To ensure that the templates were successfully immobilized, these beads were employed as templates for another PCR reaction. Amplification products were detectable in the supernatant when templates were generated from biotinylated primers, whereas no products were detected in the control reaction containing templates using non-biotinylated DNA primers (Figure 1C).

**Figure 3:**
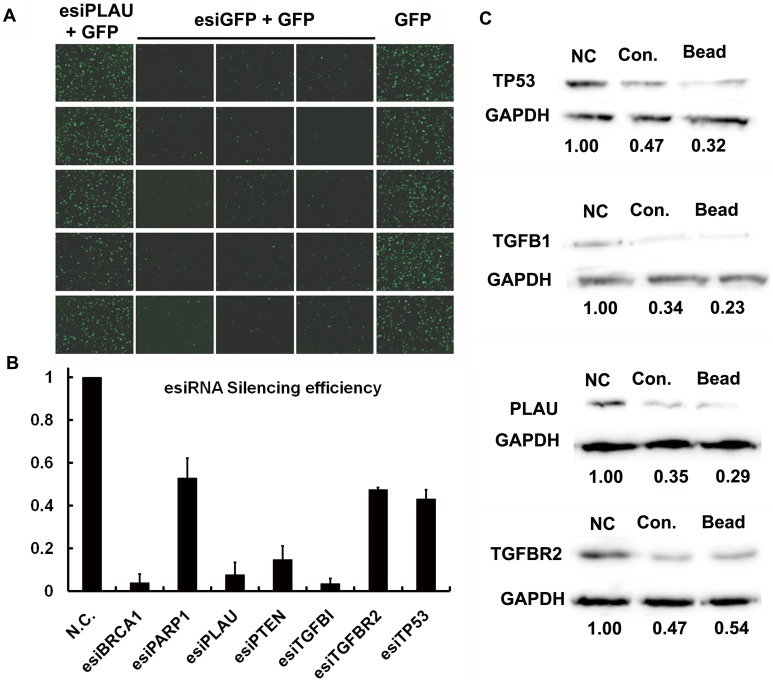
The silencing specificity and efficiency of esiRNAs. A. Fifteen GFP esiRNAs generated in parallel potently inhibited the fluorescent signal in cells cotransfected with GFP-encoding vectors; in contrast, five PLAU esiRNAs had no effect on the fluorescent intensity compared to control experiments. **B. & C.** Quantitative analysis of the silencing efficiency of esiRNA products. The qRT-PCR results showed that esiRNAs manufactured on the magnetic beads can efficiently inhibit expression levels of the respective genes. A western blot assay showed that esiRNAs targeting TP53, TGFB1, PLAU, or TGFBR2 inhibited therespective protein expression levels by up to approximately 50%.

**Figure 4:**
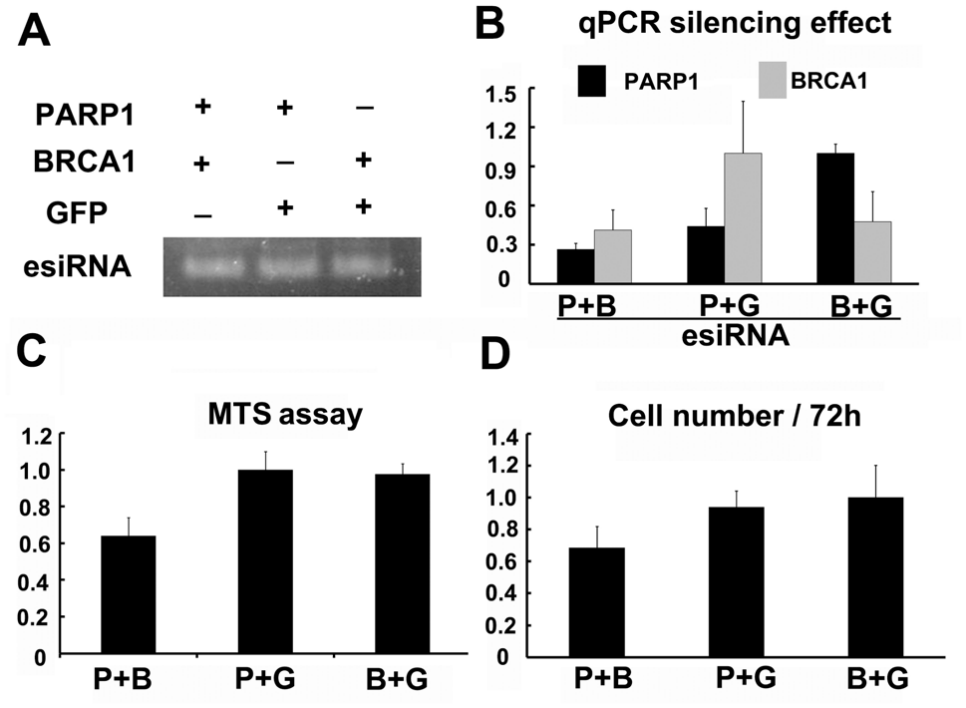
Synergistic effects of esiRNAs. **A.** The simultaneous production of two normalized esiRNAs in one well. **B.** The qRT-PCR results showed that the mixture of PARP1 and BRCA1 esiRNAs could effectively silence the expression of both genes. **C. & D.** The MTS assay and cell survival assays showed the synergistic effect of PARP1 and BRCA1 genes.

**Figure 5:**
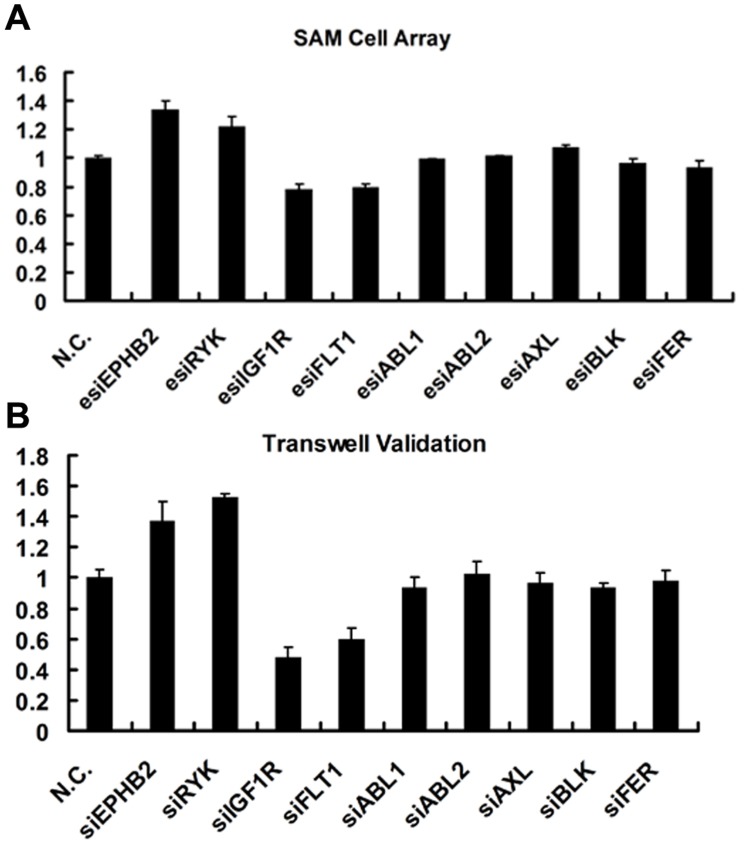
Screen and validation of tyrosine kinase genes capable of regulating the cell migration. A. Self-assembled cell microarray screen of genes capable of regulating the migration of Hela cells by use of esiRNA tyrosine kinase library. **B.** Validation of the esiRNA screen results by use of siRNAs and transwell assay.

Next, the product-immobilized microbeads were incubated with *in vitro* transcription buffer as well as T7 RNA polymerase, and large amounts of complimentary RNA strands were generated. Then, the DNA-immobilized microbeads were then removed, and a specific amount of magnetic microbeads tagged with a hybridization probe was added so that the beads would act as bait for the duplex via hybridization (Figure S1). After stringent rinsing, these RNA duplexes on the beads were enzymatically digested into esiRNAs, followed by the removal of magnetic beads. As expected, only biotinylated DNA products generated *in vitro* transcripts and subsequent esiRNA products (Figure 1C).

Removal of PCR and transcription supernatant from esiRNA products is critical, as the under- or over-digested RNA fragment contaminants would cause adverse effects on cells. Traditional purification procedures often involve centrifugation or even electrophoresis, which are both time-consuming and labor intensive in regards to large-scale level synthesis of esiRNAs. The magnetic bead-integrated chip reported here allowed quick and simple purification using a centrifuge-free approach; notably, the insufficiently digested products were removed without the need for electrophoresis or precipitation steps.

Our method allows for quantification and normalization of esiRNA products by tailoring the amount of magnetic beads in either the immobilization or hybridization step. Since the amount of transcription and digestion products mainly depended on the number of probes, we optimized the concentration of magnetic beads (Figure S2). Given the cost and yield, we chose the final concentrations of 8 fM or 0.4 pM of magnetic beads for immobilization and hybridization steps, respectively. Our results showed that a three-order magnitude difference of initial DNA template input (0.1–100 ng) could result in a variation of no more than 20% of the transcription products if the same amount of magnetic beads was added during the immobilization step (Figure 2A). The variation in the production of esiRNA became smaller (approximately 10%) if the amount of magnetic beads was further controlled during the hybridization step. In order to further confirm the reproducibility of these results, eight esiRNA products were manufactured in parallel. Less than 3% variation was observed in either the transcription or digestion products (Figure 2B). These results indicate that the amount of esiRNA products can be normalized by controlling the amount of microbeads.

To evaluate the silencing specificity and efficiency of the esiRNAs generated by our novel approach, we manufactured twenty esiRNAs in parallel, including fifteen for GFP and five for PLAU, and co-transfected each of them along with an eGFP vector into 293 T cells. The results demonstrated that only the eGFP esiRNA could silence eGFP protein expression levels, while PLAU esiRNA had no effect (Figure 3A). Next we produced seven esiRNAs, each targeting an endogenous gene, and quantitatively assessed the silencing efficiency. Quantitative RT-PCR results showed that endogenous expression levels of all genes were inhibited by up to 50% (Figure 3B). We then examined the expression level of the proteins encoded by four of the genes (TP53, TGFB1, TGFBR2 and PLAU) using western blot analysis 48 hours after transfection. We observed that esiRNAs manufactured by either our method or the traditional approach resulted in a significant silence efficiency of approximately 50% (Figure 3C).

Many cellular pathways and mechanisms are potentiated by multiple factors that work in concert to synergistically regulate downstream events. For example, cells deficient in BRCA1 were shown to be highly sensitive to additional PARP1 inhibition or knock-down, resulting in cell death via apoptosis [11], [12]. Therefore, we next investigated the ability of our approach to assess the effects of inhibiting two or more genes on certain cellular responses simultaneously. We were able to successfully manufacture three pairs of esiRNAs (PARP1 and BRCA1, PARP1 and GFP, and BRCA1 and GFP), each pair in a single well in which both DNA templates were simultaneously amplified and immobilized on magnetic beads. Our results confirmed that the presence of two templates together in a single well did not influence the production and normalization of the two esiRNAs (Figure 4A). Furthermore, quantitative RT-PCR confirmed that a mixture of PARP1 and BRCA1 esiRNAs resulted in efficient silencing of both genes (Figure 4B). Importantly, both the MTS assay and cell survival assay showed that inhibition of either PARP1 or BRCA1 did not induce significant cell death, while silencing of both genes severely impacted cell viability (Figure 4C and 4D). Thus, our chip could readily manufacture a pair of normalized esiRNAs, such as PARP1 and BRCA1, which could subsequently and immediately be used in functional analysis of their synergistic effects.

Cell migration is an essential feature of the metastasis process, and the identification and characterization of molecules that control cell migration could provide a better understanding of cancer metastasis. We used our novel magnetic bead-integrated chip approach to generate a small esiRNA library targeting 68 genes from the tyrosine kinase family. A self-assembled cell microarray, which we recently developed, allowed the high-throughput screen of functional genes regulating cell migration. Four esiRNAs targeting EPHB2, RYK, FLT1 and IGF1R were found to significantly up- or down-regulate the migration of Hela cells (Figure 5A and Table S1). To confirm this finding, we selected nine genes (including EPHB2, RYK, FLT1, and IGF1R) from the library and synthesized the siRNAs. Consistently, only the siRNAs against the four genes were found to have the same effect as the corresponding esiRNAs in influencing the migration of Hela cells (Figure 5B). These results show that the magnetic bead-integrated chip could be easily adapted to a genome-scale screen.

In summary, our study has demonstrated a convenient and robust approach for the large-scale manufacture of ready-to-use esiRNAs. This magnetic bead-integrated chip not only simplified the complicated steps in esiRNA preparation, such as amplification, transcription and enzymatic digestion, but also showed other advantages over traditional methods. First, the traditional methods involve the purification, quantification and normalization of liquid samples and require expensive instruments as well as demanding skills, which profoundly hamper the manufacture and application of esiRNAs on a large-scale level. Second, it would be a severely taxing task to quantify them one by one, as would be the case in the traditional methods. With the use of magnetic beads, we could easily separate the amplification and transcription products from buffer solutions and components. Furthermore, we could tailor the amount of esiRNA product by controlling the number of magnetic beads in the immobilization or hybridization steps. Our functional studies further showed that these esiRNAs could be immediately applied to loss-of-function studies without the need for any additional treatment. Interestingly, this approach can readily manufacture two or more normalized esiRNAs in a single well, which can be subsequently used to study the synergistic effects of the genes on cell viability. Thus, this new approach provides a more robust and cost-effective choice for manufacturing esiRNAs.

## Materials and Methods

### Preparation and Immobilization of DNA Template on Streptavidin-magnetic Beads

For each target gene, we design and obtain a double-strand DNA template of 400–500 bp in length tagged with a pair of adapters, which were used for hybridization and *in vitro* transcription. A T7 promoter sequence for *in vitro* transcription was added at each end. Thus, the entire gene sequence was: 5′-GCTCCGGA AAGCAACCCGACTAATACGACTCACTATAGG (certain target DNA) CCTATAGTGAGTCGTATTACGAGGCCTTTCG TTGGGCTG -3′. Biotinylated PCR primer and reverse primer were as follows: Forward 5′-biotin-GCTCCGGAAAGCAACC CGAC-3′ and Reverse 5′- CAGCCCAACGAAAGGCCTCG-3′.

Streptavidin (SA)-coated magnetic beads were purchased from Invitrogen (Dynal MyOne C1). Biotinylated DNA templates were immobilized on these beads following the standard protocol provided by the manufacturer.

### Fabrication of the Microchip

The microwell chip was composed of two parts, a 96-well or 384-well plate, and a magnetic mask. The latter was assembled from a group of magnetic bars so that the bar came in close contact with the well when removing the magnetic beads.

### *In vitro* Transcription and esiRNA Production on Beads

*In vitro* transcription (IVT) was carried out in reaction buffer containing T7 RNA polymerase (NEB). The magnetic beads containing immobilized DNA template were incubated with IVT buffer at 37°C for 4 h with shaking.

Once transcription finished, the DNA template immobilized magnetic beads were removed, and tag-probe immobilized magnetic beads (Invitrogen, Dynal MyOne T1) resuspended in1 X SSC were added into the supernatant. The mixture was then heated to 95°C, slowly cooled down to 65°C, and incubated at 65°C for 1 h. After these performances, dsRNA duplex would anneal and hybridize onto beads via tag-probes. Washing with 0.5×SSC, at least 3 times, would remove the excessive transcription solution and DNA templates.

The magnetic microbeads were easily removed after the transcription or the digestion step using a 96-magnetic needle plate, which was assembled with a group of electromagnetic steel needles.

Finally, following the siRNaseIII (Takara) protocol, enzymatic digestion was performed at 30°C with shaking. After 1 h, enzymatic digestion was terminated by adding EDTA. The supernantant esiRNA products were stored at −80°C until the subsequently used for transfection. The digested products were transferred into another plate for transfection with the aid of the magnetic mask.

### esiRNA Transfection, Real-time PCR, Western Blot and Cell Viability Assay

esiRNAs were transfected into 293 T or Hela cells using Lipofectamine 2000 (Invitrogen) according to the manufacture’s instruction. Cells were collected at 48 h for the real-time PCR and western blot assay, or at 72 h for the cell survival and MTS assays.

Cell lysis and protein extractions of 293 T or Hela cells were performed following the indicated procedures. Antibodies against TP53, PLAU, TGFBI, TGFBR2 and GAPDH were purchased from CST or Abcam, respectively. Sequences of qRT-PCR primers are listed in Table S2.

Real-time PCR and western blot assays were carried out following a standard protocol.The MTS Cell viability assay was performed following the instruction of CellTiter 96® AQueous Non-Radioactive Cell Proliferation Assay Kit (Promega, USA).

### Transwell Assay and Self-assembled Cell Microarray for the Cell Migration Study

For transwell migration assays, Hela cells were seeded into the upper chamber of a Transwell insert (pore size, 8 µm; Costar) in 100 µl serum-free medium per well. Medium (600 µl) containing 10% serum was added in the lower chamber to function as a chemoattractant. Non-migratory cells were removed from the upper chamber by scraping the surface with a cotton bud. The cells remaining on the lower surface of the insert were fixed with 2% formaldehyde (Sigma, USA) and stained by DAPI (Roche, USA). Self-assembled cell microarray screening assay was performed according to our previously described study [13].

## Supporting Information

Figure S1 **Schematic diagram of the process for manufacturing esiRNA using magnetic beads integrated on a chip.** GSP represents gene specific primers.
(TIF)

Figure S2 **Optimization of the concentrations of magnetic beads.** **A**. Different amounts of magnetic beads were used during the immobilization step. The transcription products were normalized. **B**. Different amounts of Tag-probe immobilized beads were added before the hybridization step. The yield of esiRNA products was normalized.
(TIF)

Table S1 **Migration assay using the esiRNA tyrosine kinase library.** The esiRNAs were manufactured using the magnetic bead-integrated chip approach. Samples 1, 2 and 3 represent three different esiRNAs all targeting one gene, respectively.
(DOC)

Table S2 **Sequences of qRT-PCR primers used in the experiments.**
(DOC)
