# Supplementary figures and images for: A Magnetic Bead-Integrated Chip for the Large Scale Manufacture of Normalized esiRNAs
Source: PLoS One. 2012 Jun 27;7(6):e39419. doi: 10.1371/journal.pone.0039419 (PMC3384639; doi:10.1371/journal.pone.0039419)

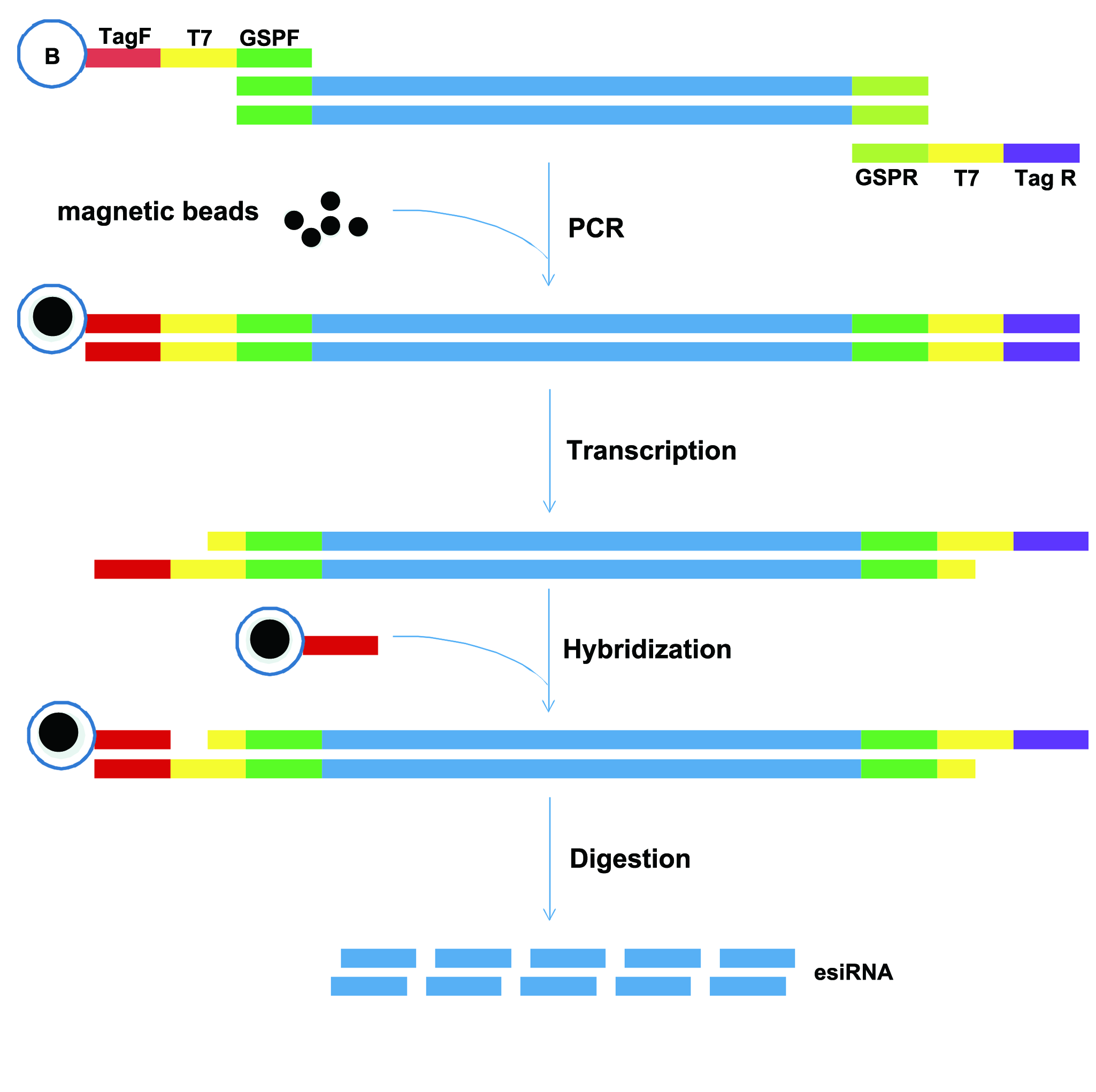

Supplement: Figure S1 — Schematic diagram of the process for manufacturing esiRNA using magnetic beads integrated on a chip. GSP represents gene specific primers. (TIF) [file pone.0039419.s001.tif]

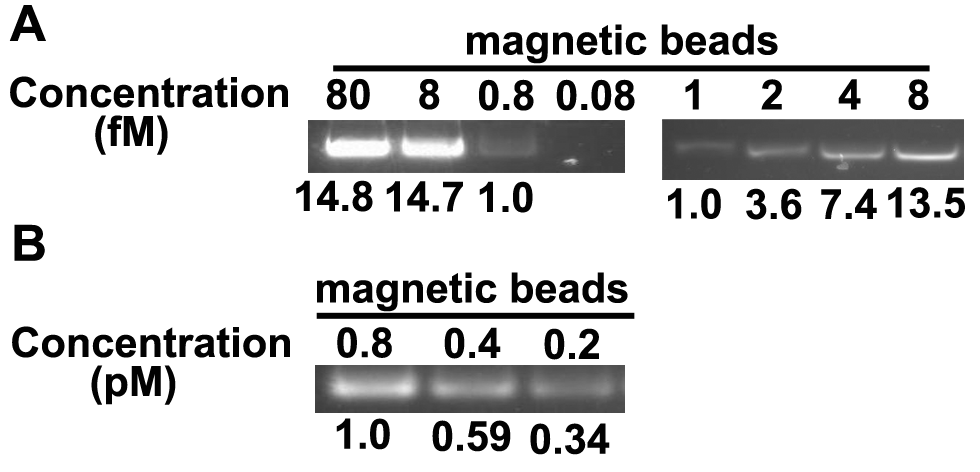

Supplement: Figure S2 — Optimization of the concentrations of magnetic beads. A. Different amounts of magnetic beads were used during the immobilization step. The transcription products were normalized. B. Different amounts of Tag-probe immobilized beads were added before the hybridization step. The yield of esiRNA products was normalized. (TIF) [file pone.0039419.s002.tif]
