# Supplementary material for: A Magnetic Bead-Integrated Chip for the Large Scale Manufacture of Normalized esiRNAs
Source: PLoS One. 2012 Jun 27;7(6):e39419. doi: 10.1371/journal.pone.0039419 (PMC3384639; doi:10.1371/journal.pone.0039419)
Supplement: Table S1 — Migration assay using the esiRNA tyrosine kinase library. The esiRNAs were manufactured using the magnetic bead-integrated chip approach. Samples 1, 2 and 3 represent three different esiRNAs all targeting one gene, respectively. (DOC) [file pone.0039419.s003.doc]

**Table S1. Screen of tyrosine kinase genes capable of regulting the migration of Hela cells by use of SAMcell**.

| esiRNA | Sample 1 | Sample 2 | Sample 3 | Average |
| --- | --- | --- | --- | --- |
| IGF1R | 0.78 | 0.74 | 0.82 | 0.78 |
| FLT1 | 0.78 | 0.78 | 0.83 | 0.80 |
| FGFR1 | 0.90 | 0.92 | 0.90 | 0.91 |
| EPHB6 | 0.92 | 0.93 | 0.90 | 0.92 |
| EPHA2 | 0.91 | 0.93 | 0.93 | 0.92 |
| FER | 0.98 | 0.90 | 0.94 | 0.94 |
| ROS1 | 0.98 | 0.91 | 0.94 | 0.95 |
| FGFR4 | 0.98 | 0.94 | 0.92 | 0.95 |
| EGFR | 0.99 | 0.93 | 0.92 | 0.95 |
| FGFR2 | 0.89 | 0.99 | 0.96 | 0.95 |
| PTK7 | 0.92 | 0.94 | 0.98 | 0.95 |
| DDR1 | 0.93 | 0.95 | 0.97 | 0.95 |
| EPHB3 | 0.92 | 0.95 | 0.98 | 0.95 |
| SRC | 0.89 | 1.02 | 0.94 | 0.95 |
| YES1 | 1.03 | 0.94 | 0.89 | 0.95 |
| TEK | 0.94 | 0.95 | 0.99 | 0.96 |
| EPHA6 | 0.94 | 0.98 | 0.96 | 0.96 |
| FES | 0.89 | 1.04 | 0.95 | 0.96 |
| LTK | 1.02 | 0.95 | 0.93 | 0.97 |
| BLK | 0.94 | 0.99 | 0.97 | 0.97 |
| HCK | 0.99 | 0.92 | 0.99 | 0.97 |
| PDGFRA | 0.99 | 0.97 | 0.95 | 0.97 |
| RET | 0.95 | 0.99 | 0.98 | 0.97 |
| DDR2 | 1.04 | 0.95 | 0.93 | 0.97 |
| SYK | 1.02 | 0.92 | 0.99 | 0.97 |
| EPHA5 | 0.93 | 1.04 | 0.96 | 0.98 |
| TXK | 1.05 | 0.98 | 0.91 | 0.98 |
| LCK | 0.94 | 0.98 | 1.03 | 0.98 |
| ITK | 0.92 | 1.05 | 0.99 | 0.99 |
| EPHA7 | 0.94 | 0.98 | 1.04 | 0.99 |
| MET | 1.08 | 0.91 | 0.98 | 0.99 |
| ABL1 | 0.99 | 0.99 | 0.99 | 0.99 |
| ZAP70 | 0.95 | 1.02 | 1.02 | 1.00 |
| FLT3 | 1.00 | 0.99 | 1.00 | 1.00 |
| LYN | 1.00 | 1.02 | 0.97 | 1.00 |
| FLT4 | 1.01 | 1.04 | 0.94 | 1.00 |
| TYRO3 | 1.02 | 1.05 | 0.92 | 1.00 |
| BMX | 1.00 | 1.00 | 1.00 | 1.00 |
| ROR1 | 1.02 | 0.99 | 1.00 | 1.00 |
| BTK | 1.00 | 1.02 | 0.99 | 1.00 |
| PDGFRB | 1.02 | 0.94 | 1.05 | 1.00 |
| TEC | 0.98 | 1.02 | 1.01 | 1.00 |
| PTK6 | 1.02 | 1.01 | 0.98 | 1.01 |
| JAK2 | 0.98 | 0.99 | 1.05 | 1.01 |
| ROR2 | 1.01 | 1.03 | 0.98 | 1.01 |
| TTK | 0.98 | 0.99 | 1.05 | 1.01 |
| MUSK | 1.02 | 0.99 | 1.01 | 1.01 |
| JAK1 | 0.95 | 1.03 | 1.05 | 1.01 |
| FGFR3 | 1.03 | 0.95 | 1.05 | 1.01 |
| JAK3 | 0.96 | 1.05 | 1.02 | 1.01 |
| EPHA8 | 0.98 | 1.04 | 1.02 | 1.02 |
| ABL2 | 1.02 | 1.02 | 1.02 | 1.02 |
| EPHA1 | 1.02 | 1.02 | 1.02 | 1.02 |
| EPHA4 | 1.04 | 1.04 | 0.99 | 1.02 |
| CSK | 1.03 | 1.03 | 1.03 | 1.03 |
| EPHB1 | 1.04 | 1.05 | 1.01 | 1.04 |
| FRK | 1.14 | 0.92 | 1.05 | 1.04 |
| TIE1 | 1.02 | 1.02 | 1.07 | 1.04 |
| EPHB4 | 1.03 | 1.09 | 1.01 | 1.04 |
| FYN | 1.02 | 1.10 | 1.02 | 1.05 |
| INSR | 1.02 | 1.04 | 1.10 | 1.06 |
| KIT | 1.04 | 1.10 | 1.05 | 1.06 |
| MERTK | 1.07 | 1.10 | 1.02 | 1.06 |
| KDR | 1.08 | 1.03 | 1.11 | 1.07 |
| AXL | 1.06 | 1.08 | 1.09 | 1.08 |
| EPHA3 | 1.09 | 1.09 | 1.07 | 1.08 |
| RYK | 1.28 | 1.24 | 1.16 | 1.23 |
| EPHB2 | 1.34 | 1.40 | 1.29 | 1.34 |

The esiRNAs used in this experiment is manufactured by use of a magnetic bead-integrated chip. Sample 1, 2 and 3 represent three esiRNAs targeting one gene. Value for each sample referred to the ratio of the cell areas at 0 and 9 hours, which then was normalized by control samples targeting GFP.
