# Supplementary material for: A Magnetic Bead-Integrated Chip for the Large Scale Manufacture of Normalized esiRNAs
Source: PLoS One. 2012 Jun 27;7(6):e39419. doi: 10.1371/journal.pone.0039419 (PMC3384639; doi:10.1371/journal.pone.0039419)
Supplement: Table S2 — Sequences of qRT-PCR primers used in the experiments. (DOC) [file pone.0039419.s004.doc]

**Table S2. Sequences of qRT-PCR primers.**

| Gene | Forward primer | Reverse primer |
| --- | --- | --- |
| DGCR8 | CAAGCAGGAGACATCGGACAAG | CACAATGGACATCTTGGGCTTC |
| TP53 | TCAACAAGATGTTTTGCCAACTG | ATGTGCTGTGACTGCTTGTAGATG |
| ING1 | CAACAACGAGAACCGTGAGA | GAGCGCTTCTTCTTCTTGGA |
| TGFBR2 | GGAAGCTCATGGAGTTCAGC | CAGCAGCTCTGTGTTGTGGT |
| PLAU | GTCTACCTGGGTCGCTCAAG | CACAGCATTTTGGTGGTGAC |
| TGFB1 | GCGTGCTAATGGTGGAAAC | CGGTGACATCAAAAGATAACCAC |
| PTEN | AACCCACCACAGCTAGGAACT | ATACACATAGCGCCTCTGAC |
| PARP1 | GAGGTGGATGGGTTCTCTGA | CTTGGCATACTCTGCTGCAA |
| BRCA1 | AGGACAAAGCAGCGGATACA | TTCATCCCTGGTTCCTTGAG |
| CDK5 | GTCCATCGACATGTGGTCAG | GTCATAGAGGGCCACTGCTC |
| GAPDH | GCACCGTCAAGGCTGAGAAC | GCCTTCTCCATGGTGGTGAA |
